# Whey Protein Supplementation Enhances Whole Body Protein Metabolism and Performance Recovery after Resistance Exercise: A Double-Blind Crossover Study

**DOI:** 10.3390/nu9070735

**Published:** 2017-07-11

**Authors:** Daniel W. D. West, Sidney Abou Sawan, Michael Mazzulla, Eric Williamson, Daniel R. Moore

**Affiliations:** 1 Faculty of Kinesiology and Physical Education, University of Toronto, Toronto, ON M5S 1A1, Canada; daniel.west@utoronto.ca (D.W.D.W.); sidney.abousawan@mail.utoronto.ca (S.A.S.); m.mazzulla@mail.utoronto.ca (M.M.); eric.williamson@mail.utoronto.ca (E.M.); 2 Kinesiology and Physical Education University of Toronto 100 Devonshire Place, Toronto, ON M5S 2C9, Canada

**Keywords:** net protein balance, dietary protein, ergogenic aid, strength, power

## Abstract

No study has concurrently measured changes in free-living whole body protein metabolism and exercise performance during recovery from an acute bout of resistance exercise. We aimed to determine if whey protein ingestion enhances whole body net protein balance and recovery of exercise performance during overnight (10 h) and 24 h recovery after whole body resistance exercise in trained men. In a double-blind crossover design, 12 trained men (76 ± 8 kg, 24 ± 4 years old, 14% ± 5% body fat; means ± standard deviation (SD)) performed resistance exercise in the evening prior to consuming either 25 g of whey protein (PRO; MuscleTech 100% Whey) or an energy-matched placebo (CHO) immediately post-exercise (0 h), and again the following morning (~10 h of recovery). A third randomized trial, completed by the same participants, involving no exercise and no supplement served as a rested control trial (Rest). Participants ingested [^15^N]glycine to determine whole body protein kinetics and net protein balance over 10 and 24 h of recovery. Performance was assessed pre-exercise and at 0, 10, and 24 h of recovery using a battery of tests. Net protein balance tended to improve in PRO (*P* = 0.064; effect size (ES) = 0.61, PRO vs. CHO) during overnight recovery. Over 24 h, net balance was enhanced in PRO (*P* = 0.036) but not in CHO (*P* = 0.84; ES = 0.69, PRO vs. CHO), which was mediated primarily by a reduction in protein breakdown (PRO < CHO; *P* < 0.01. Exercise decreased repetitions to failure (REP), maximal strength (MVC), peak and mean power, and countermovement jump performance (CMJ) at 0 h (all *P* < 0.05 vs. Pre). At 10 h, there were small-to-moderate effects for enhanced recovery of the MVC (ES = 0.56), mean power (ES = 0.49), and CMJ variables (ES: 0.27–0.49) in PRO. At 24 h, protein supplementation improved MVC (ES = 0.76), REP (ES = 0.44), and peak power (ES = 0.55). In conclusion, whey protein supplementation enhances whole body anabolism, and may improve acute recovery of exercise performance after a strenuous bout of resistance exercise.

## 1. Background

Consuming a source of protein after resistance exercise is essential to maximize muscle protein synthesis and net protein balance [1,2], both of which are required to support muscle hypertrophy with training. Current research supports the consumption of a moderate dose (~20–25 g) of rapidly digested, leucine-rich proteins to optimize muscle protein synthesis [3,4,5]; this ostensibly positions whey protein as a valuable supplemental source for individuals aiming to maximize their recovery from and adaptation to resistance exercise. Individuals who train at night due to preference and/or lifestyle may be particularly sensitive to nutrition interventions given that muscle and whole body protein balance is negative during the overnight period in the absence of dietary protein [6,7]. Res and co-workers [7] recently reported that ingesting 40 g of casein before sleep improved whole body protein synthesis and net balance, and enhanced muscle protein synthesis during overnight recovery compared to a carbohydrate placebo. Post-exercise/pre-bedtime protein ingestion for evening exercisers may also translate into greater increases in muscle strength and hypertrophy with chronic training and protein consumption [8]. Thus, research to date points toward the importance of protein feeding to enhance protein accretion after resistance exercise, especially if normal dietary patterns preclude the ability to eat during prolonged (e.g., 8–12 h) overnight recovery in evening exercisers. Whether rapidly digested whey protein, also enhances anabolism, similar to slowly digested casein, and exercise recovery when consumed after an evening training bout remains to be determined.

Athletes aiming to maximize lean mass growth and post-exercise recovery would ostensibly benefit from enhancing whole body anabolism. The use of oral tracers such as [^15^N]glycine have a long history of use in noninvasively measuring free-living whole body protein metabolism in a variety of populations (for an extensive review of the end-product method of measuring protein turnover and its historical use, see [9]). This method can be used to measure whole body protein synthesis, protein breakdown, and net protein balance over both shorter (i.e., ~10 h) and longer (i.e., ~24 h) time frames [10], which highlights its utility in determining protein metabolism over early and later post-exercise recovery periods in controlled yet free-living scenarios (e.g., [11,12,13]). Moreover, the noninvasive nature of the tracer would minimize any residual effects of the tracer methodology (e.g., muscle biopsies) on subsequent performance tests. While measures of whole body protein metabolism cannot delineate an effect in any one tissue, nutritionally-mediated changes in whole body net protein balance have been shown to qualitatively mirror that of myofibrillar protein synthesis over relatively prolonged (i.e., 12 h) post-exercise recovery periods [14,15]. Therefore, individuals who can maximize whole body net protein balance would likely also support greater skeletal muscle remodeling, which may persist for up to 24–48 h after an acute bout of exercise [16,17].

Strenuous training can result in changes in muscle function characterized by acute impairments in strength and exercise performance over the subsequent hours-to-days after a training bout [18,19]. While this acute loss of performance, potentially mediated by exercise-induced muscle damage [19,20], may be considered a normal byproduct of any training program, the rapid resolution of these negative effects could ultimately facilitate a higher quality training stimulus during in-season trainig and/or sport performance. Whereas the beneficial effect of protein supplementation on chronic muscular adaptations have been investigated extensively [12,21,22,23], little research has addressed whether post-exercise protein ingestion may facilitate the acute (e.g., ≤24 h) recovery of exercise performance. One study [24] reported improved force-generating capacity in sedentary men over a 24-h recovery period after whey hydrolysate ingestion. However, it is unclear whether these findings are similar in trained athletes and/or when whey is consumed after an evening bout of exercise. Furthermore, studies examining the relationship between exercise recovery and protein ingestion post-exercise tend to use a between-group [24,25] rather than a repeated-measures experimental design, and sometimes without controlling for diet [24,26]. Both of these factors are potentially important in order to draw meaningful conclusions regarding the effect of a nutritional supplement towards enhancing performance [27]. Therefore, additional research is warranted to determine to what extent protein supplementation may facilitate a more rapid recovery of muscle performance after an acute bout of resistance exercise. Thus, in the present study, we employed a crossover design, controlled diet, and conducted a battery of tests to assess the recovery of exercise performance.

The primary aim of the present study was to determine if consuming a whey protein supplement post-exercise enhances whole body net protein balance over a 10 h overnight recovery period. Moreover, given the unequal distribution of dietary protein typical of Western diets [28], a secondary aim was to determine if the greater 10 h overnight response could be sustained over 24 h in a free-living environment by supplementing the breakfast meal with a second protein supplement. Finally, given the ability of whey protein to enhance post-exercise rates of muscle protein synthesis, which would presumably enhance the repair of exercise-induced muscle damage, we also conducted performance tests to assess the recovery of muscle strength and endurance as well as anaerobic power and neuromuscular function over 10 and 24 h of recovery. We hypothesized that protein supplementation would enhance net protein balance at 10 and 24 h of recovery, primarily by enhancing protein synthesis, and that this response would be associated with greater indices of exercise performance.

## 2. Materials and Methods

### 2.1. Experimental Protocol

Twelve healthy young men (76 ± 8 kg, 24 ± 4 years old, 14% ± 5% body fat; means ± standard deviation (SD)), who were resistance training two to four times per week for at least six months, provided written consent to participate in a protocol that was written in accordance with standards set by the revised (2008) Declaration of Helsinki, and that was approved by the research ethics board at the University of Toronto, Toronto, Canada (protocol # 32576). Additional self-reported inclusion criteria to participate were as follows: non-smoking, no supplement consumption for at least 3 weeks prior to the trial’s commencement, and no medication that may affect protein metabolism (e.g., corticosteroids or non-steroidal anti-inflammatories). Participants completed a Physical Activity Readiness Questionnaire ([29], revised 2002 version) in order to help confirm that they could safely perform the exercise protocol. After an overnight fast, air displacement plethysmography (BOD-POD, COSMED USA Inc., Chicago, IL, USA) was used characterize participant lean mass, fat mass, and body composition (Supplemental Table S1).

### 2.2. Carbohydrate and Protein Supplemented Trials

**Figure 1:**
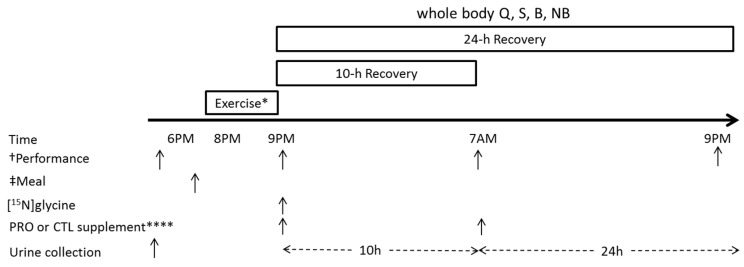
A schematic representation of the trial day. Participants were free-living in recovery and consumed a controlled diet that mimicked their habitual dietary intake. * Exercise: on supplemented trials only; whole body, heavy resistance exercise. † Isometric maximal voluntary contraction, squat jump, Wingate test, knee extension repetitions to failure at 75% of 1-repetition maximum. ‡ Mixed-macronutrient meal. A twenty-five gram (25 g) whey protein supplement (PRO) or isocaloric carbohydrate (control; CTL) supplement. Q, nitrogen turnover; S, whole body protein synthesis; B, whole body protein breakdown; NB, whole body net protein balance. Urine collection was collected over two intervals: 0–10 h, and 10–24 h; after obtaining a sample from the 0–10 collection, both collections were pooled to obtain a 24 h recovery sample.

In a double-blind placebo-controlled crossover fashion, participants performed a strenuous bout of whole body resistance exercise in the evening prior to consuming one serving of MuscleTech 100% Whey protein (PRO) or an energy-matched placebo (CHO). The supplements were consumed immediately after exercise as well as the following morning (i.e., after 10 h of recovery). One serving of the protein supplement contained 25 g of whey protein (a blend of whey peptides, isolates, and concentrates), 2.5 g fat, and 3 g carbohydrate, yielding ~130 kcal of energy. The supplements were consumed on each of the two trial days (two trial days per condition; see Figure 1) in addition to controlled diets (described below). At the beginning of each trial, participants completed a performance test and then consumed a mixed-macronutrient meal (~18:00), which was a standardized proportion of their daily controlled diet: 28% kcal, 20% protein, 31% carbohydrate, and 31% fat. Participants then completed the remainder of the given trial (i.e., whole-body resistance exercise at 20:00, according to Figure 1). Trial randomization, study blinding, and supplement preparation were performed by an individual who was external to the study. Each trial was separated by approximately one week.

### 2.3. Rest Trial

A third randomized trial involving no exercise and no supplement served as a rested control trial (Rest), and controlled for potential time-of-day effects in performance testing [30]. All of the aspects of the rested trial were identical to the supplemented trials described above, except that participants rested (sat quietly) in the laboratory instead of performing a whole body resistance exercise bout and consuming a supplement.

### 2.4. Dietary Controls

Prior to the study trials, participants completed 3-day diet logs. The diet logs were analysed using ESHA (Elizabeth Stewart Hands and Associates, company founders) Research Food Processor Nutrition Analysis Software (Salem, OR, USA). Prepackaged diets were prepared by a registered dietician, and provided energy requirements estimated using the Harris–Benedict equation and a moderate activity factor (1.5). Participants were provided with four individualized meals on each of the two trial days for each of the three conditions (Rest, CHO, PRO). The protein content provided in the controlled diets was equal to habitual intake, and was evenly distributed between the four meals. The carbohydrate content provided was 4–5 g/kg/day. Fat provided the balance of total energy. A food checklist was completed by each participant to track dietary compliance. Pilot data from our lab estimated that the energy expended as a result of the whole body exercise used in the present study was ~200 kcal. Given that each supplement (protein or carbohydrate placebo) provided after the exercise bout contained 130 kcal of energy, participants consumed an additional 70 kcal of energy in the form of a protein-free cookie [31] to help achieve energy balance during overnight recovery.

### 2.5. Exercise

Participants were familiarized with performance test protocols and the whole body resistance exercise bout approximately one week before the first trial. Participants’ three repetition maximum (RM) strength was determined for the exercises to be performed in the whole body exercise trials. The exercise bout (CHO and PRO trials only) consisted of supersets of barbell bench press and pulldown superset, and barbell overhead press and seated row, respectively, as well as leg press and leg extension (isolated). For all of the supersets or isolated exercises, participants performed 4 sets of 10 reps at 75% of their 1 repetition maximum (RM) with 2 min rest intervals between sets. Participants were asked to refrain from exercise in the 48 h period prior to the start of each trial, as well as during the 24 h intervention period.

### 2.6. Performance Testing

Exercise performance was assessed pre-exercise, and at 0, 10, and 24 h of recovery (Figure 1). On Rest, all of the aspects of the protocol were maintained (e.g., the timing of performance testing), except that participants rested instead of performing the resistance exercise bout. For each trial, participants reported to the lab at 17:30. After a 3 min treadmill warm-up at a self-selected pace, participants completed four performance tests in the following order: countermovement jump (CMJ), knee extension isometric maximal voluntary contraction (MVC), repetitions to failure at 75% of 1 RM (REP), and a 30 s Wingate test. These tests were used to assess neuromuscular fatigue [32], static [33,34] and dynamic strength/muscular endurance, and anaerobic power [35], respectively. Verbal encouragement from the investigators was provided to participants for all of the tests. Three minutes rest were given after the countermovement jump and MVC tests, and 5 min rest were given between REP and the Wingate test.

#### 2.6.1. Countermovement Jump

Participants performed three countermovement jumps on a force plate (Advanced Mechanical Technology Inc. (AMTI), Watertown, MA, USA) with 60 s rest between repetitions. To begin each test, participants stepped on the force plate and were asked to stand still briefly (~3 s), before instruction from the investigator to jump when they were ready. Participants were instructed to descend to a comfortable depth before jumping as high as possible (no pauses were required, and arm swing was permitted). Force plate output signals were amplified and converted (AMTI GEN 5 Amplifier, Watertown, MA, USA) to a digital ground reaction force for each millisecond over the 10 s collection period. Jump variables were analysed from ground reaction force data in Excel, in a manual and blinded fashion, using previously described criteria/calculations [32].

#### 2.6.2. Isometric Maximal Voluntary Contraction

Unilateral knee extensor MVC was assessed using a custom dynamometer in which participants were seated with their legs secured to a pad that was coupled to a strain gauge. Force output signals were recorded using PowerLab with LabChart Pro v.8.0.5 (ADInstruments Inc., Colorado Springs, CO, USA). The knee was positioned at a 90° angle, and aligned with the axis of rotation. Participants performed three warm-up contractions (25–75% of maximal effort) prior to three maximal 5 s contractions, separated by 60 s of rest between repetitions. The best of the three scores was used in the analysis.

#### 2.6.3. Dynamic Repetitions to Failure

Participants performed as many knee extension repetitions as possible at 75% of 1 RM. Repetition counts were standardized using the maximal height to which participants could lift the weight by fully extending their legs; repetitions below this cut-off were omitted.

#### 2.6.4. Wingate

Participants performed a 30 s maximal Wingate test on a stationary bicycle (Monark Ergomedic 839 E; Monark Exercise AB, Vansbro, Sweden). After performing a brief warm-up, the tests were initiated once participant pedalling reached 140 revolutions per minute. Participants pedalled as fast as they could against a resistance set to 7.5% of their body weight for 30 s; data was recorded using Monark Anaerobic Test Software v3.3 (Monark Exercise AB, Vansbro, Sweden).

### 2.7. Stable Isotope and Urine Analysis

To determine whole body nitrogen turnover, [^15^N]glycine (2 mg/kg body weight, Cambridge Isotope Inc., Andover, MA, USA) was dissolved in 200 mL of water before oral consumption after exercise and immediately before supplement consumption (or corresponding time during Rest). ‘Spot’ urine samples, obtained upon arrival to the lab prior to each trial, were used to determine baseline enrichments. The urine was collected over two consecutive intervals for each trial: 21:00–07:00 (10 h overnight recovery) and 07:00–21:00 the day after the trial (pooled with urine produced in the 10 h recovery, for 24 h analyses). The urine was collected in plastic containers containing glacial acetic acid as a chemical preservative, and was kept at 4 °C before being aliquoted and stored at −80 °C until analysis. The creatinine concentration was measured using a QuantiChrom Creatinine Assay Kit (cat. DICT-500, BioAssay Systems, Hayward, CA, USA). The urea concentration was measured using a QuantiChromTM Urea Assay Kit (cat. DIUR-500, BioAssay Systems, Hayward, CA, USA). Coefficients of variation for the creatinine and urea assays were 5.4% and 2.8%, respectively. Isotopic enrichments of urea and ammonia were analysed in duplicate by isotope-ratio mass spectrometry (Metabolic Solutions Inc., Nashua, NH, USA). Whole body nitrogen turnover (Q) was calculated from urinary nitrogen end-products, ammonia and urea, as follows:

(1) $$Q\left(\mathrm{ammonia}\right)=\left(\frac{d}{Time\;10h\;t:Tr-0h\;t:Tr}\right)÷body\;mass\times time$$

(2) $$Q\left(\mathrm{urea}\right)=\left(\frac{d}{Time\;24h\;t:Tr-0h\;t:Tr}\right)÷body\;mass\times time$$

where d is the oral ^15^N dose (g glycine × 0.1972), t:Tr is the tracer-to-tracee ratio of ammonia or urea. Q over 10 h was calculated as per Q(ammonia) above. Q over 24 h was calculated as the harmonic mean:

(3) $$Q\left(24\;h\right)=\frac{1}{2}\times \frac{Q\left(\mathrm{ammonia}\right)\times Q\left(\mathrm{urea}\right)}{Q\left(\mathrm{ammonia}\right)+Q\left(\mathrm{urea}\right)}$$

Whole body protein synthesis was calculated as:

(4) $$\mathrm{Synthesis}\;=\left(Q-\left(N_{Cr}+N_{\mathrm{urea}}+N_{\mathrm{misc}}\right)\right)\times 6.25$$

where N_Cr_ is N excretion from creatinine, N_urea_ is N excretion from urea, and N_misc_ is miscellaneous nitrogen excretion (estimated at 0.5 mg N/kg body wt/h).

Whole body protein breakdown was calculated as:

(5) $$\mathrm{Breakdown}=\left(Q-\frac{\mathrm{total}\;N\;\mathrm{intake}}{\mathrm{body}\;\mathrm{mass}}\right)\times 6.25$$

Whole body net protein balance was calculated as:

Net balance = Synthesis − Breakdown
(6)

### 2.8. Sample Size and Statistics

**Sample size.** Our primary outcome was whole body net protein balance over 10 h of recovery. Previous research has demonstrated that pre-bedtime protein feeding increases whole body net protein balance relative to a protein-free control using intravenous tracer administration [7]; here, we aimed to determine whole body net balance by oral [^15^N]glycine tracer. Providing a 25 g dose of whey protein in PRO results in ~0.3 g protein/kg body weight (assuming an average body mass of 80 kg) difference in protein intake over the acute 10 h recovery period compared to a protein-free isocaloric control. This difference in protein intake is similar to that which was previously reported to result in a greater whole body net protein balance over 9 h (as determined by oral [^15^N]glycine) in children who consumed ~0.32 g/kg protein immediately after exercise compared to a group that consumed a protein-free control [13]. Therefore, the study by Moore et al. [13] utilizing a cross-over design is the most similar from a methodological and intervention standpoint to the present study, and was used to power our study to detect a difference in the primary outcome. In the study by Moore et al. [13], the whole body net protein balance in the high protein (HP) group over 9 h was 46.8 mg/kg/h and the control group was 18.5 mg/kg/h, with an average standard deviation of 22.3 mg/kg/h. With α = 0.05, β = 0.8, and utilizing a between participant comparison (to be conservative given any unknown child–adult differences with this methodology), *n* = 10 participants were determined to be sufficient to detect a significant difference between PRO and CHO (according to: [36]). To account for a potential 20% drop out rate, we recruited *n* = 12 participants.

**Statistics.** Protein metabolism data at 10 and 24 h were calculated using different end-product pools (see 2.7 above), which contributed to unequal variance between time points. However, because the data were normally distributed within each time point, and because the primary outcome of interest at each time point was between-condition differences, protein metabolism data were analyzed by one-way repeated measures analysis of variance (condition: Rest, CHO, PRO) at 10 h and 24 h. One-way repeated measures (time: Pre, 0 h; CHO and PRO only) analysis of variance (ANOVA) was also used to determine if performance was impaired at 0 h, due to the exercise bout in PRO and CHO. Tukey’s post hoc was used to determine significant (*P* < 0.05) pairwise differences. The analysis of variance and post hoc analyses were conducted using SigmaStat version 3.1 software (Systat Software, Point Richmond, CA, USA). While conventional ‘*P*-value statistics’ are well-suited to determine if something is false by rejecting a zero-effect hypothesis, effect size calculations have been recommended for data analyses in the sports sciences because the magnitude of an effect is not confounded by the sample size, is not prone to false-discovery conclusions that may occur due to multiple comparisons, and often helps the investigator/reader to make a practical conclusion [37,38]. Thus, effect size analysis was planned a priori during the conception and design of the study protocol with Cohen’s *d* effect sizes (ES, [39]) and the Probability of Protein Superiority being calculated to assess whether protein supplementation affected protein metabolism and the rate of exercise performance recovery over 10 and 24 h post-exercise. Effect sizes were calculated using the standard deviation from the Rest trial [40,41]. Percent (%) probability of PRO Superiority (PS) versus CHO was calculated from Cohen’s *d* as follows: % = φ (*d*/$\sqrt{2}$), where φ is the cumulative distribution function of the standard normal distribution, and *d* is the Cohen Effect Size [42]. If there was no effect of protein supplementation—i.e., CHO and PRO distribution curves show 100% overlap—PS would equal 50%. An exploratory analysis was also performed with Pearson correlations of whole body net protein balance with percent recovery of exercise performance and habitual protein intake over 10 and 24 h of recovery.

## 3. Results

### 3.1. Participant Characteristics and Study Controls

#### 3.1.1. Participant Characteristics

Participant characteristics are shown in Supplemental Table S1.

#### 3.1.2. Dietary Intake on Trial Days

Participants completed compliance logs to record the food that they consumed during the 2-day controlled diet period (all food was provided). According to the logs, compliance was high, with participants consuming 98.4% of the energy and 98.4% of the protein that was provided in the diets.

#### 3.1.3. Study Blinding

Given that the performance testing involved in this study and potential placebo effects, it was important that the participants were effectively blinded to the supplement condition. For the 24 trials involving supplement consumption, participants answered ‘(C) I don’t know’ ten times to a questionnaire asking whether they believed they received ‘(A) a protein supplement’, ‘(B) a carbohydrate supplement’, or ‘(C) I don’t know’. Of the remaining 14 times that participants responded (A) or (B), they guessed correctly 9 times and incorrectly 5 times (on average, 7 correct guesses would be expected merely due to chance). Only 1 of 12 participants correctly identified both CHO and PRO supplements. Thus, we have good reason to believe that the participants were well-blinded and that knowledge of the supplement condition was not a factor in the study’s outcomes.

### 3.2. Protein Metabolism

**Figure 2:**
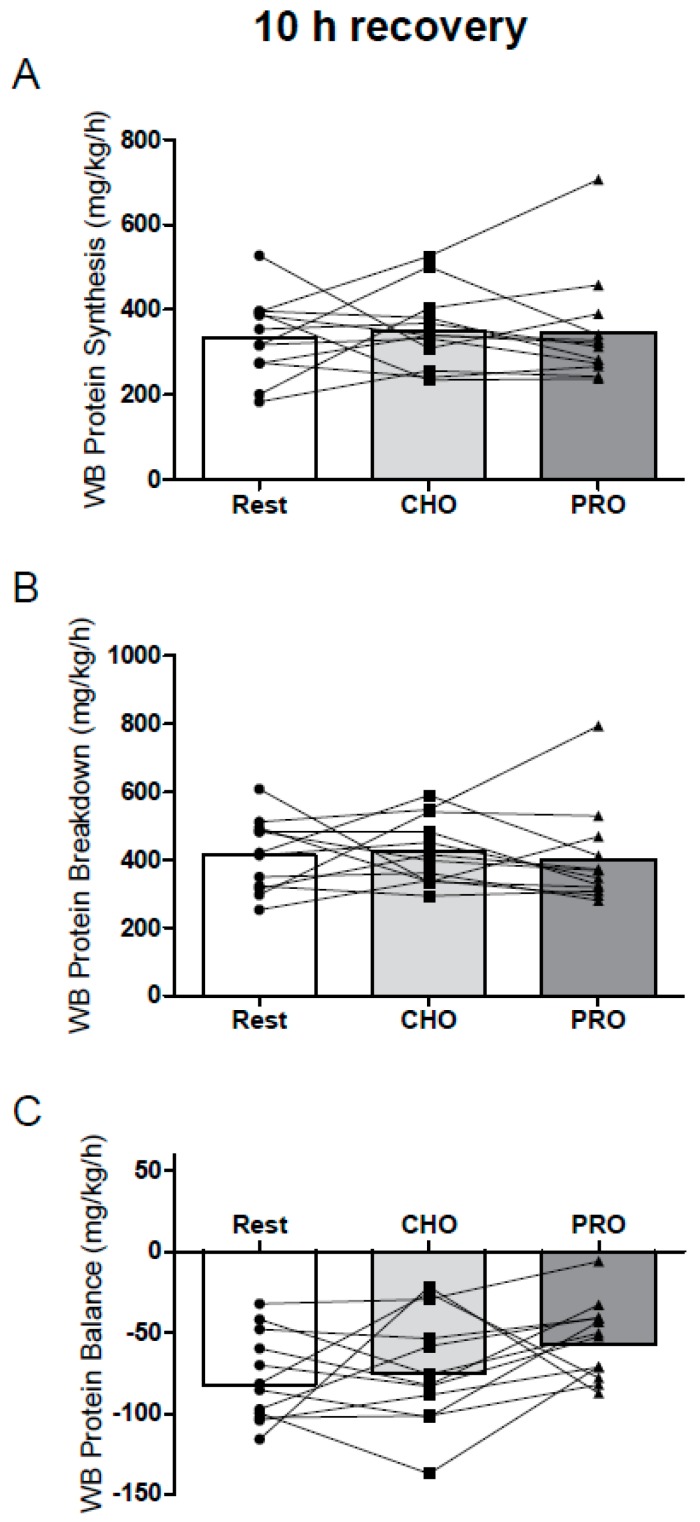
10 h whole body (WB) protein synthesis (**A**), protein breakdown (**B**), and protein balance (**C**), at rest (Rest) and after whole body resistance exercise supplemented with 25 g whey protein (PRO) and isocaloric carbohydrate (CHO), calculated using urinary [^15^N]ammonia end product enrichment. *P* = 0.064 for one-way (condition) repeated-measures ANOVA of whole body protein balance. Values are individual means; *n* = 12. WB, whole body.

**Figure 3:**
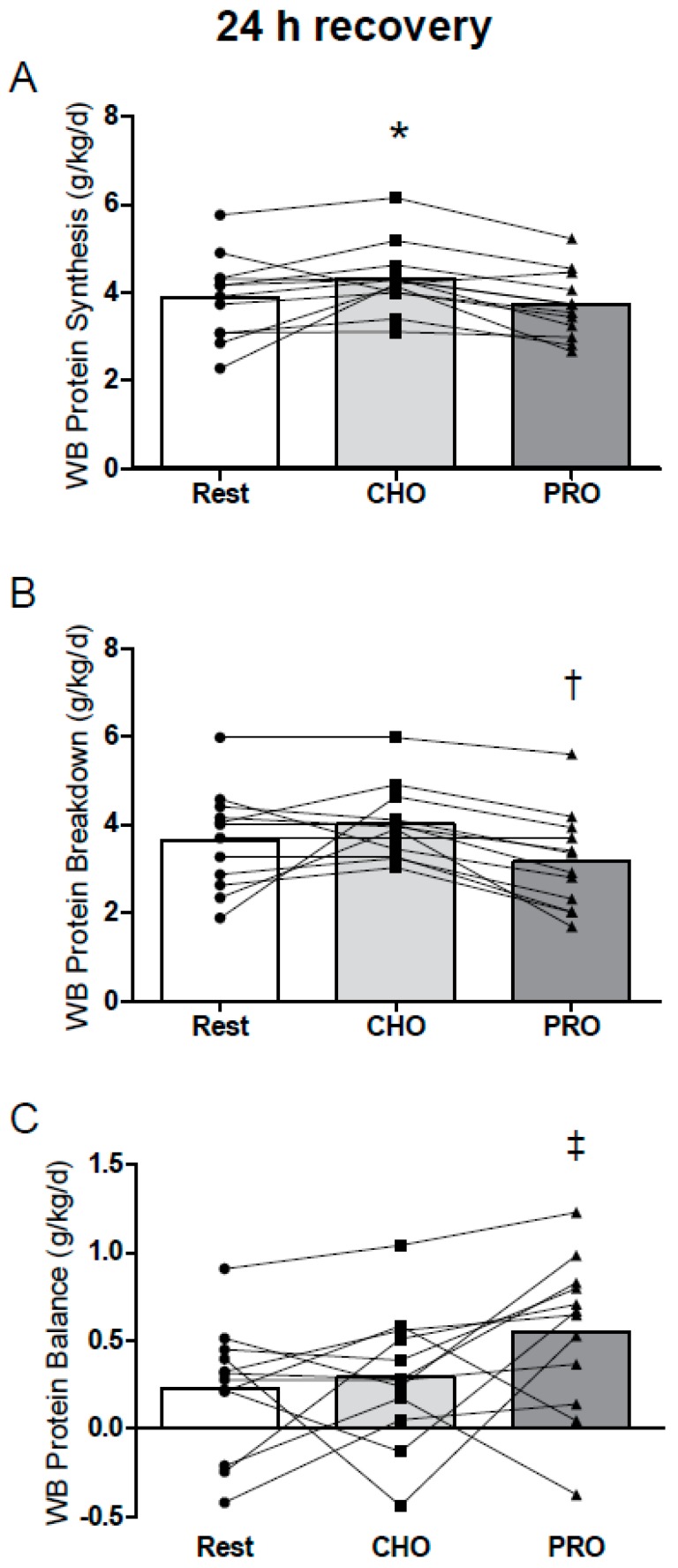
24 h whole body (WB) protein synthesis (**A**), protein breakdown (**B**), and protein balance (**C**) at rest (Rest) and after whole body resistance exercise supplemented with 25 g whey protein (PRO) and isocaloric carbohydrate (CHO), calculated using the harmonic mean of urinary [^15^N]ammonia and urea end product enrichments. Data were analysed by one-way (condition) repeated-measures ANOVA: * CHO > PRO, *P* = 0.017; *P* = 0.11 for CHO vs. Rest. Protein breakdown: † PRO < CHO, *P* = 0.006. Net protein balance: ‡ PRO > Rest, *P* = 0.036; *P* = 0.11 for PRO vs. CHO. Values are individual means; *n* = 12.

At 10 h of recovery, there were no major effects of exercise or protein supplementation on whole body nitrogen turnover or protein synthesis or breakdown (Figure 2A,B). Net protein balance was negative during overnight recovery (all groups), and there was a trend toward an effect of condition on net protein balance during overnight recovery (*P* = 0.064; ES = 0.61; PS = 67%; Figure 2C). Over 24 h of recovery, whole body protein synthesis was greater after CHO (Figure 3A), whereas protein breakdown was suppressed in PRO (Figure 3B); the net effect was a protein balance that was enhanced in PRO (*P* = 0.036 vs. Rest; ES = 0.69; PS = 69%; Figure 3C) but not in CHO (*P* = 0.84 vs. Rest).

#### Correlation of Net Protein Balance and Habitual Dietary Intake

We correlated the net protein balance with habitual dietary protein intake to examine whether individuals’ different habitual protein intakes had differential protein losses (in the overnight fasted state) or gains (24 h recovery). The net protein balance was negatively correlated with habitual dietary protein intake at 10 h recovery in CHO and PRO (*r* = −0.64 and −0.68, respectively both *P* < 0.05), but only in CHO at 24 h recovery (*r* = 0.69, *P* = 0.013), not PRO (*r* = 0.43, *P* = 0.16).

### 3.3. Exercise Performance Recovery

All of the performance data percent differences and 90% CI are summarized in Supplemental Table S2. As expected, the bout of whole body resistance exercise decreased (all *P* < 0.05 by one-way (time) ANOVA) performance at 0 h across all tests: MVC (~20%), repetitions to failure (~19%), peak and average anaerobic power (both ~7%), and CMJ height (~12%).

#### 3.3.1. Recovery of Maximal Strength, Muscle Endurance, and Anaerobic Power

Effect sizes and the Probability Protein Superiority for MVC, REP, and the Wingate tests are shown in Table 1. At 10 h of recovery, there were small-to-moderate beneficial effects of protein supplementation for MVC and mean anaerobic power during the Wingate. At 24 h of recovery, there was a moderate beneficial effect of protein supplementation on MVC, REP, and peak power during the Wingate.

#### 3.3.2. Recovery of Countermovement Jump Performance

The CMJ results are presented in Table 2. Briefly, there was a moderate beneficial effect of protein supplementation on jump height at 10 h, which was associated with moderate improvements in the following CMJ variables: maximum rate of force development, eccentric (pre-load) velocity, peak power, and eccentric and total duration. At 24 h, movement through the eccentric (pre-load) phase still favoured PRO, as indicated by a greater peak eccentric velocity, a shorter eccentric duration, and a greater eccentric phase force-velocity area-under-the-curve. However, these superior eccentric phase variable profiles were accompanied by a longer (compared to CHO) concentric duration, which decreased average force in the concentric phase, and likely nullified the force potential developed in the eccentric phase from carrying forward to the final jump height.

#### 3.3.3. Correlation of Net Protein Balance and Performance

We correlated whole body net protein balance with the percent recovery of performance outcomes to explore whether the moderate performance effects that we observed in PRO were associated with changes in net protein balance. No correlations were apparent for MVC, REP, or Wingate peak or mean power with net protein balance at 10 h (Pearson *r* < 0.30 and *P* > 0.15 for all correlations) or at 24 h (Pearson *r* < 0.30 and *P* > 0.25 for all correlations).

## 4. Discussion

The main finding of the present study was that whey protein, but not carbohydrate, supplementation after a bout of resistance exercise in the evening enhanced whole body net protein balance over 10 h and 24 h of recovery compared to a rested control day. In contrast to our hypothesis, we did not observe a statistically greater whole body net protein balance with protein supplementation over the acute 10 h period compared to a carbohydrate control, which may reflect the unexpected response variation in our outcome measure. Nevertheless, magnitude-based statistics revealed there were moderate beneficial effects of protein supplementation relative to a protein-free isocaloric control to enhance early (i.e., 10 h) and later (i.e., 24 h) acute whole body anabolism during the immediate 24 h post-exercise recovery period. Additionally, we present data that suggests that whey protein supplementation enhances the rate of acute performance recovery in trained young men. Specifically, there were moderate beneficial effects of protein supplementation for enhanced maximal strength, anaerobic power, and neuromuscular function at 10 h, and maximal strength, anaerobic power, and repetitions to failure at 24 h of recovery.

### 4.1. Effect of Protein Ingestion on Net Protein Balance

Although recovery is clearly multi-faceted, individuals who intend to facilitate their recovery from an exercise stimulus and/or promote subsequent adaptation should ostensibly aim to enhance whole body protein anabolism. The accretion of lean body mass with resistance training is ultimately underpinned by acute exercise-induced increases in net protein balance that, over time, summate to changes in lean body mass [12,43]. Prof. van Loon’s group was the first to report that protein ingestion (in the form of slowly digested casein) before bed enhances muscle protein synthesis, whole body protein synthesis, and net protein balance during the overnight, post-exercise recovery period [7,44]. The lack of any apparent effect on whole body protein synthesis or breakdown over the 10 h recovery period in our hands appears at odds with that of Res and colleagues from the van Loon group [7], but is nonetheless consistent with previous research with this oral tracer [13]. Importantly, the lack of any detectable change in protein kinetics does not preclude the possibility that subtle changes in synthesis and/or breakdown may still translate into meaningful differences in whole body net protein balance. Here, we show that whey protein tends to improve overnight whole body net protein balance, and demonstrates a moderately beneficial effect relative to an isocaloric control.

We have previously demonstrated that [^15^N]glycine end-product derived rates of whole body net protein balance (see ref. [9] for a review of the method) generally align with the rates of myofibrillar protein synthesis over 12 h of recovery [14,15], and qualitatively predict training-induced increases in lean body mass when measured over 24 h [12]. Thus, the greater net protein balance after protein ingestion in the present study was likely associated with enhanced rates of muscle protein synthesis, which is consistent with previous work demonstrating that pre-bedtime protein ingestion increases muscle protein synthesis and tracer-derived rates of whole body net protein balance [7]. Interestingly, whereas whole body net balance was greater over 24 h with whey protein ingestion, this appeared to be facilitated in part by a reduction of whole body protein breakdown. This could suggest that the combination of 25 g (~0.32 g/kg) of protein in the morning in addition to a moderate breakfast protein intake (~0.48 g/kg) resulted in a saturation in muscle protein synthesis, but also an attenuation in whole body protein breakdown to maximize anabolism, as previously observed [45]. In contrast, the greater whole body protein synthesis over 24 h with carbohydrate intake is likely a reflection of enhanced rates of non-muscle protein synthesis, given that: (i) carbohydrate (and the associated insulin response) does not enhance muscle protein synthesis with adequate protein ingestion [46,47]; and (ii) this 24 h increase in whole body protein synthesis was not associated with greater whole body net protein balance, which would be expected to occur in concert with enhanced rates of muscle protein synthesis [14,15]. Regardless, supplementing a balanced diet with a rapidly digested protein source such as whey protein can enhance overnight and 24 h whole body anabolism and, if sustained chronically, may enhance anabolic signaling [48] and training-induced lean mass accretion [21,49].

### 4.2. Impact of Habitual Protein Intake on Net Protein Balance

The observation of generally greater whole body protein balance with post-exercise whey protein ingestion is consistent with a postprandial stimulation of muscle and whole body anabolism after whey protein ingestion [50,51]. Although whole body anabolism was greater with protein ingestion, the single 25 g dose was insufficient to generate a net positive protein balance over this overnight recovery period. This may be related to the relatively high (i.e., ~1.9 g/kg/day) habitual protein intake of our participants, which, although slightly higher than current recommendations for athletes [52], is generally consistent with reported intakes in strength athletes [43]. Indeed, there was a negative correlation between habitual intake and overnight net protein balance, which is consistent with a previous study showing greater fasted losses at higher protein intakes [53]. The 25 g dose of whey protein (~0.32 g/kg) provided ~17% of the participants’ habitual intake for this macronutrient, which is less than the ~45% (~0.54 g/kg) provided to participants in the study by Res and colleagues [7]. Notwithstanding differences in tracer methodology and protein type between the studies, these data could collectively suggest that individuals who have higher habitual protein intakes may benefit from a greater protein dose and/or more repeated protein feedings after evening exercise to optimize whole body anabolism. In support of this notion, the negative relationship between protein intake and whole body net balance was sustained over 24 h with the carbohydrate supplement, but was absent in the protein condition, which benefited from an additional 25 g of whey protein feeding in the morning. Therefore, whereas muscle protein synthesis is maximized with the ingestion of ~20–25 g of high quality protein [4,5], more frequent protein feedings may be required to replenish fasted state losses and maximize whole body protein balance [15].

### 4.3. Effect of Whey Supplementation on Exercise Performance Recovery

In addition to protein metabolism ‘growth’ outcomes, we were interested in examining whether whey supplementation impacted the rate of exercise performance recovery. Intense resistance exercise that involves an eccentric component often induces muscle damage that can manifest as increased muscle soreness and/or impaired muscle function [18,19,20]. In theory, protein supplement-facilitated improvements in net protein balance may promote muscle remodelling and speed the recovery of muscle function [26], which in turn could improve the quality of subsequent training/performance demands. Milk-based protein supplementation immediately after a bout of damaging exercise (i.e., maximal lengthening contractions) has been shown to attenuate decrements in muscle strength and repeated sprints 24–72 h after exercise [54,55,56]. Our study extends these findings, by demonstrating that whey protein ingestion can enhance muscle performance, as observed through the beneficial effects on maximal isometric force and anaerobic mean power, as early as 10 h into recovery. Moreover, we report beneficial effects of whey protein supplementation that extend up to 24 h into recovery with improvements in repetitions to failure, peak aerobic power, and maximal strength; these findings are consistent with the beneficial effects of milk-protein consumption over similar recovery periods [56]. Given that impaired strength is a hallmark of muscle damage [20], the protein supplement may have facilitated a more rapid restoration of muscle function through a greater remodelling of the force-generating myofibrillar protein pool [5,57]. Although speculative, greater myofibrillar remodelling would ostensibly be consistent with an improved recovery of anaerobic power in the protein compared to the carbohydrate supplemented condition at 24 h post-exercise. Collectively, our results suggests that whey protein ingestion after evening exercise and the following morning may improve muscle reconditioning following exercise, and may be advantageous for those aiming to enhance the recovery of force generation and maintain training quality [27].

The countermovement jump test is of practical importance for athletes and provides a reasonable reflection of neuromuscular function [32,58,59], which can be impaired by resistance exercise-induced muscle damage [60]. Gathercole and colleagues have recently demonstrated that monitoring the force, velocity, and power outcomes of the CMJ, as well as neuromuscular/movement strategy variables (e.g., duration of various CMJ phases), can reveal fatigue-induced neuromuscular changes for 24 h or longer [32,58]. In the present study, there was a moderate effect for jump height to be greater with protein supplementation at 10 h but not 24 h of recovery, suggesting that acute post-exercise protein ingestion may facilitate a more rapid restoration of neuromuscular function when the recovery duration is limited (e.g., <10 h). Examining CMJ variables revealed that post-exercise protein ingestion tended to improve the maximum rate of force development at 10 h of recovery (ES = 0.72). Moreover, whereas the concentric phase appeared to be slightly enhanced at 10 h and hindered at 24 h, there was a moderate beneficial effect of protein supplementation on eccentric phase variables at both 10 h and 24 h. These findings are generally aligned with a previous observation [55] that ~34 g of milk protein consumed immediately after a bout of muscle damaging exercise (i.e., maximal lengthening contractions) improves reactive strength 48 h into recovery. Therefore, our data suggest that whey protein supplementation can aid in the recovery of an explosive functional movement after an acute bout of high-volume resistance exercise, and may have additional relevance for athletes competing in high intensity stop-and-go sports [56].

The mechanisms underpinning the generally greater markers of exercise performance with whey protein ingestion are not yet elucidated; however, it has previously been suggested that a greater muscle protein repair/remodeling subsequent to enhanced rates of protein synthesis may facilitate a more rapid performance recovery with protein supplementation [27]. Previous work has suggested that the beneficial effects of protein supplementation on the recovery of force production are independent of muscle damage and oxidative stress [61]. However, some evidence suggests that essential amino acids may attenuate inflammation/muscle soreness [62,63] and muscle damage [64] after exercise; if this were to be the case in the present study, then it could have positively impacted all performance tests, including the CMJ movement strategy (pre-load velocity was higher, and eccentric phase shorter, with protein supplementation).

### 4.4. Enhancements in Whole Body Net Protein Balance and Performance Were Not Correlated

Inasmuch as the greater net protein balance in the present study may reflect greater rates of muscle protein remodelling, we performed correlations to determine whether participants with a higher net protein balance also experienced superior performance recovery. We observed no association between 10 and 24 h whole body net protein balance and change of performance (relative to post-exercise), suggesting there was no evidence of a ‘responder’ phenotype that had concomitantly enhanced whole body protein accretion and exercise performance recovery. This observation may be related to the fact that, due to its slow rate of turnover, skeletal muscle contributes ~30% of whole body protein balance [65], which may have precluded our ability to model with sufficient precision the net muscle protein balance from our whole body tracer. Alternatively, it is possible that a dose-response relationship for muscle and/or whole body protein balance towards performance recovery does not exist, in which case inducing some undefined minimum net anabolism is sufficient to enhance and/or maximize performance recovery. However, we are aware of no other studies that have concomitantly measured protein metabolism (either at the muscle or whole body level) and acute performance recovery in strength-trained athletes that could yield insight to the present results. Thus, whereas protein ingestion may be a viable strategy for those aiming to enhance exercise performance, determining the mechanism(s) for this effect requires further research that includes measures of both protein metabolism and muscle function. Nevertheless, our results support the concept that whey protein supplementation after an evening training session [7,8] could potentially support greater training adaptations [21,27] through an enhancement of whole body net protein balance (present results and [7,8]) and/or greater training quality/volume due to a more rapid recovery of exercise performance (present study and [8]).

## 5. Conclusions

In summary, the consumption of 25 g of whey protein after an evening bout of resistance exercise tended to improve whole body net protein balance over 10 h of overnight recovery compared to a rested control, and was moderately beneficial versus an isocaloric carbohydrate post-exercise supplement. Consuming an additional 25 g of whey protein in the morning after exercise contributed to the maintenance of a greater whole body protein balance over the 24 h recovery period compared to a rested control and carbohydrate supplementation. The greater whole body anabolism with whey protein supplementation was also associated with enhanced recovery exercise performance after an intense bout of resistance exercise. Collectively, our data suggest that resistance-trained individuals may benefit from protein supplementation after an evening bout of resistance exercise as well as the following morning to attenuate overnight fasted-state protein losses and enhance exercise performance recovery.

## Figures and Tables

**Table 1:** Exercise performance recovery effect sizes: REx + CHO vs. REx + PRO.

	Cohen Effect Size		Probability of Protein Superiority	
Outcome	10 h recovery	24 h recovery	10 h recovery	24 h recovery
*Protein metabolism*				
Net protein balance	0.61	0.69	67%	69%
*Knee extension*				
Peak isometric force	0.28	0.76	58%	70%
Repetitions to failure	0.11	0.44	53%	62%
*Wingate test*				
Peak power	0.27	0.55	58%	65%
Mean power	0.49	0.12	64%	53%

Unless indicated otherwise, effect sizes were calculated as the mean difference between PRO and CHO divided by the standard deviation (SD) of Rest (control) [40]. The thresholds for Small, Moderate and Large effect sizes are 0.2, 0.5 and 0.8, respectively. [39]. Probability of protein superiority: the percent chance that a value from PRO will be greater than CHO, calculated as % = φ (*d*/$\sqrt{2}$), where φ is the cumulative distribution function of the standard normal distribution, and *d* is the Cohen Effect Size [42]; 50% = no effect. REx = whole body resistance exercise.

**Table 2:** Neuromuscular fatigue effect sizes: REx + CHO vs. REx + PRO.

	Cohen Effect Size		Probability of Protein Superiority	
CMJ Outcome	10 h recovery	24 h recovery	10 h recovery	24 h recovery
Jump height	0.49	−0.29	64%	42%
*Force*				
Mean force (CON)	−0.04	−0.56	49%	35%
Max RFD	0.72	0.12	69%	53%
Total impulse (CON)	0.36	−0.01	60%	50%
Peak force	−0.07	−0.08	48%	48%
Force-Velocity AUC (ECC)	0.48	0.56	63%	65%
*Velocity*				
Peak velocity	0.27	−0.05	58%	48%
Take-off velocity	0.29	−0.09	58%	48%
Mean velocity (CON)	0.27	−0.05	58%	48%
Kinetic energy at take-off	0.29	−0.09	58%	47%
Peak ECC (pre-load) velocity	0.54	0.49	65%	64%
*Power*				
Peak power	0.24	−0.38	58%	39%
Time to peak power	0.58	0.28	66%	58%
*Neuromuscular strategy*				
Concentric duration	−0.29	00.94	42%	25%
Eccentric duration	0.56	0.45	65%	62%
Total duration	0.56	0.26	65%	57%

Effect sizes were calculated as the mean difference between PRO and CHO divided by the SD of Rest (control) [40]. The thresholds for Small, Moderate and Large effect sizes are 0.2, 0.5 and 0.8, respectively [39]. Beneficial effects are shown as mathematically positive for all CMJ variables (e.g., a positive effect size is shown for a shorter CMJ duration). Probability of protein superiority: the percent chance that a value from PRO will be greater than CHO, calculated as % = φ (*d*/$\sqrt{2}$), where φ is the cumulative distribution function of the standard normal distribution, and *d* is the Cohen Effect Size [42]; 50% = no effect. CMJ, countermovement jump; CON, concentric; ECC, eccentric. RFD = rate of force development. AUC = area-under-the-curve. Impulse is the area under the force-time curve. REx = whole body resistance exercise.

## Supplementary Materials

The following are available online at www.mdpi.com/2072-6643/9/7/735/s1, Table S1: Participant anthropometric and habitual dietary characteristics; Table S2: Post-exercise performance recovery. All data generated or analysed during this study are included in this published article and its supplementary information files.
