# Supplementary material for: Whey Protein Supplementation Enhances Whole Body Protein Metabolism and Performance Recovery after Resistance Exercise: A Double-Blind Crossover Study
Source: Nutrients. 2017 Jul 11;9(7):735. doi: 10.3390/nu9070735 (PMC5537849; doi:10.3390/nu9070735)
Supplement: Supplementary file 1 [file nutrients-09-00735-s001.zip › nutrients-206383-supplementary.pdf]

Supplemental Table S1. *Participant anthropometric and habitual dietary characteristics*

Participant characteristics	
Anthropometrics	
Age (y)	24.3 ± 4.5
Height (m)	1.76 ± 0.6
Body weight (kg)	76.2 ± 8.0
Body fat (%)	14.3 ± 4.6
Fat-free mass (kg)	65.1 ± 6.2
**Dietary characteristics**	
Protein (g/d)	143 ± 16
Relative protein (g/kg/d)	1.9 ± 0.3
Carbohydrate (g/d)	366 ± 63
Fat (g/d)	78 ± 11
Total energy (kcal)	2734 ± 220
% of total energy	
Protein	21 ± 2
Carbohydrate (g/d)	53 ± 9
Fat (g/d)	26 ± 4

Supplemental Table S2. Post-exercise performance recovery

	% change from post-exercise (90% confidence interval)							
	**10 h recovery**				**24 h recovery**			
	CHO		PRO		CHO		PRO	
	% Δ	(90% CI)	% Δ	(90% CI)	% Δ	(90% CI)	% Δ	(90% CI)
Knee extension								
Peak isometric force	4.3	(-0.5 to 9.2)	9.7	(2.9 to 16.5)	15.1	(10.4 to 19.7)	25.0	(15.6 to 34.3)
Repetitions to failure*	0.7	(-0.6 to 2.0)	1.0	(-0.2 to 2.1)	2.3	(0.3 to 4.3)	3.3	(0.9 to 5.7)
Wingate test								
Peak power	-1.3	(-3.4 to 0.7)	0.6	(-3.3 to 4.4)	3.9	(-2.1 to 9.9)	9.3	(4.0 to 14.6)
Mean power	-1.4	(-3.6 to 0.9)	0.3	(-3.5 to 4)	4.4	(0.8 to 8.1)	5.3	(2.1 to 8.6)
**CMJ Outcome**								
Jump height	1.1	(-3.1 to 5.3)	3.9	(0.8 to 6.9)	10.1	(5.6 to 14.5)	8.6	(4.5 to 12.8)
Force								
Mean force (CON)	-2.2	(-0.1 to -4.3)	-2.4	(-3.3 to -1.5)	2.7	(-0.5 to 5.8)	-0.3	(-1.7 to 1.0)
Max RFD	1.6	(-13.2 to 16.4)	15.6	(-10.4 to 41.6)	12.7	(-7.1 to 32.4)	15.2	(-2.8 to 32.9)
Total impulse (CON)	-3.7	(-6.4 to -1.1)	1.2	(-5.5 to 7.9)	3.8	(-1.0 to 8.6)	3.8	(-2.4 to 10.0)
Peak force	-1.3	(-4.2 to 1.5)	-1.8	(-3.9 to 0.3)	1.4	(-2.4 to 5.3)	0.8	(-2.4 to 4.0)
Force-Vel. AUC (ECC)	-4.9	(-15.7 to 5.8)	3.7	(-9.8 to 17.3)	-1.0	(-12.9 to 10.9)	9.6	(-7.5 to 26.8)
Velocity								
Peak velocity	-2.3	(-6.3 to 1.6)	1.9	(-5.7 to 9.6)	4.4	(-1.4 to 10.2)	3.8	(-3.0 to 10.6)
Take-off velocity	-1.9	(-5.8 to 2.0)	2.9	(-5.1 to 10.9)	5.6	(-0.5 to 11.7)	4.6	(-2.7 to 11.8)
Mean velocity (CON)	-3.8	(-8.5 to 0.9)	0.4	(-7.2 to 8.1)	5.8	(-1.3 to 12.9)	2.9	(-3.2 to 9.0)
Peak ECC (pre-load) velocity	-1.8	(-6.2 to 2.7)	3.1	(-4.5 to 10.8)	-0.8	(-5.0 to 3.4)	4.3	(-0.7 to 9.3)
Power & Kinetic Energy								
Peak power	-2.6	(-6.4 to 1.2)	1.1	(-6.7 to 9)	6.2	(-0.2 to 12.5)	2.6	(-4.1 to 9.4)
Time to peak power	3.5	(-0.6 to 7.6)	-2.9	(-7.3 to 1.6)	1.3	(-5.6 to 8.1)	-1.9	(-6.9 to 3.1)
Kinetic energy at take-off	0.96	(0.89 to 1.02)	1.06	(0.91 to 1.21)	1.12	(1.01 to 1.23)	1.11	(0.96 to 1.26)
Neuromuscular strategy								
Concentric duration	0.4	(-3.6 to 4.4)	5.0	(-0.9 to 10.8)	-2.1	(-6.9 to 2.6)	6.8	(-0.1 to 13.7)
Eccentric duration	3.0	(-2.0 to 7.9)	-4.6	(-11.4 to 2.1)	2.5	(-5.0 to 10.0)	-4.7	(-11.5 to 2.1)
Total duration	2.7	(-1.0 to 6.3)	-3.0	(-7.2 to 1.2)	0.6	(-5.8 to 7.0)	-2.2	(-6.9 to 2.6)
